# ﻿A new combination and a new synonym of Gesneriaceae in China

**DOI:** 10.3897/phytokeys.232.108644

**Published:** 2023-09-15

**Authors:** Zheng-Long Li, Zhang-Jie Huang, Da-Wei Chen, Xin Hong, Fang Wen

**Affiliations:** 1 Anhui Provincial Engineering Laboratory of Wetland Ecosystem Protection and Restoration, School of Resources and Environmental Engineering, Anhui University, CN-230601, Hefei City, Anhui Province, China Guangxi Institute of Botany, Guangxi Zhuang Autonomous Region and Chinese Academy of Sciences Guilin China; 2 Guangxi Key Laboratory of Plant Conservation and Restoration Ecology in Karst Terrain, Guangxi Institute of Botany, Guangxi Institute of Botany, Guangxi Zhuang Autonomous Region and Chinese Academy of Sciences, CN-541006, Guilin City, Guangxi Zhuang Autonomous Region, China Anhui University Hefei China; 3 Key Laboratory of Resource Biology and Biotechnology in Western China, Ministry of Education, Northwest University, CN-710069, Xi’an, Shaanxi Province, China Northwest University Xi'an China; 4 Gesneriad Committee of China Wild Plant Conservation Association, National Gesneriaceae Germplasm Resources Bank of GXIB, The Gesneriad Conservation Center of China, Guilin Botanical Garden, Chinese Academy of Sciences, CN-541006 Guilin, Guangxi, China Guilin Botanical Garden, Chinese Academy of Science Guilin China; 5 Yunnan Key Laboratory for Integrative Conservation of Plant Species with Extremely Small Populations, Kunming Institute of Botany, Chinese Academy of Sciences, CN-650201, Kunming, Yunnan, China Kunming Institute of Botany, Chinese Academy of Sciences Kunming China

**Keywords:** *Didymocarpus*, Flora of China, Gesneriaceae, new combination, new synonym, *Petrocodon*, taxonomy

## Abstract

*Didymocarpussubpalmatinervis* W.T.Wang was shown to be conspecific with *Petrocodonlithophilus* Y.M.Shui, W.H.Chen & Mich.Möller, by checking original literature, examining specimens, tracing specimen collecting history, and conducting field surveys. The results show morphological characteristics and geographical distribution overlaps between these two species. The transfer of *Didymocarpussubpalmatinervis* to *Petrocodon* as a new combination with *Petrocodonsubpalmatinervis* (W.T.Wang) F.Wen & Z.L.Li is proposed here, and *P.lithophilus* is synonymized with *P.subpalmatinervis*. Color photographs and essential information are also provided, including a detailed comparison of description, distribution, habitat, and the proposed IUCN conservation status.

## ﻿Introduction

*Didymocarpus* Wall. has a complex taxonomic history, saturated with doubtful taxa (Weber and Burtt 1998). Originally, in the taxonomic system of Burtt and Wiehler (1995), it was the largest genus in the tribe Didymocarpeae Endl., subfam. Cyrtandroideae, with more than 180 species. Weber and Burtt (1998) excluded the section Heteroboea, which was considered as part of *Didymocarpuss. l* (Hilliard and Burtt 1995; Weber and Burtt 1998). In addition, they considered that 1) *D.hancei* Hemsl. (endemic to China) and *D.bonii* Pellegr. (distributed in Vietnam and East Thailand) should be included in *Calcareoboea* C.Y.Wu ex H.W.Li, as well as *D.mollifolius* W.T,Wang and *D.niveolanosus* D.Fang & W.T.Wang; 2) *D.demissus* Hance should be assigned to the previous genus, *Chirita* Buch.-Ham. ex D. Don (Wei et al. 2010; Wang et al. 2011; Weber et al. 2011). Their notion of separating *D.hancei* from *Didymocarpus* Wall. was supported by Li (2007) based on morphological and molecular evidence. Weber et al. (2011) published three new combinations in their revision of the genus *Petrocodon* Hance, viz., *Petrocodonhancei* (Hemsl.) A.Weber & Mich.Möller (≡*Didymocarpushancei* Hemsl.), *Petrcodonmollifolius* (W.T.Wang) A.Weber & Mich.Möller (≡*Didymocarpusmollifolius* W.T.Wang) and *Petrocodonniveolanosus* (D.Fang & W.T.Wang) A.Weber & Mich.Möller (≡*Didymocarpusniveolanosus* D.Fang & W.T.Wang). *D.subpalmatinervis* W.T.Wang, a species with no collection record after 1905, also has morphological characteristics placing it in section Heteroboea. The taxonomic status of this species remained unchanged because of the difficulty in obtaining type materials. Its problematic taxonomic status has previously been recognized by (Li et al. 2015; Möller et al. 2016; Hong et al. 2018).

The French Catholic priest Francois Ducloux (1864–1945), the head of the Kunming Church from 1889 to 1945, hired people to collect plant specimens extensively in central and northern Yunnan (Qu 2014). In 1905, Ducloux collected a Gesneriaceae-like taxon, perennial and acaulescent in Y-dje, near Lou-lan, Yunnan, China (collection number *Fr. Ducloux 3711*). He sent three sheets back to the Museum of Natural History in Paris. Since then, those specimens were neglected in P for over 90 years, until in 1996, Wen-Tsai Wang identified Ducloux’s specimens as a new species belonging to Didymocarpussect.Heteroboea and published it as *Didymocarpussubpalmatinervis* subsequently a year later (Weitzman et al. 1997).

*Petrocodonlithophilus* was described at the morphological and molecular level from Naigu Stone Forest, Yunnan Province, where Michael Möller and Yu-Min Shui first discovered it in August 2012. It is placed in *Petrocodon* by some obvious characters of this genus, *viz.* a rhizomatous rosette with leaves usually rounded or elliptic, mainly in an alternate arrangement, filaments straight, stigma discoid, and fruits dehiscing loculicidally into two valves (Chen et al. 2014).

When sorting out the specimens of Didymocarpussect.Heteroboea of, the type specimen of *D.subpalmatinervis* (*Fr. Ducloux 3711*), morphologically, was found to be extremely similar to *Petrocodonlithophilus*. According to protologue and label information on the type specimen, the type locality of *Didymocarpussubpalmatinervis* is near Lunan Stone Forest, only 13 kilometers away from Naigu Stone Forest. During field investigations near the type locality of *Petrocodonlithophilus*, several populations of Didymocarpusaff.subpalmatinervis were also found on the hills. The authors concluded that *D.subpalmatinervis* is conspecific with *Petrocodonlithophilus*. Accordingly, it is reasonable to make a new combination *P.subpalmatinervis*, and reduce *P.lithophilus* to a synonym.

## ﻿Materials and methods

A thorough comparison of the type material of *Petrocodonlithophilus* and *Didymocarpussubpalmatinervis* was made. Their protologues and relevant records were studied intensively. The geographical distribution of the two species was outlined by careful field surveys of the type locality areas. Classical plant taxonomic methods were involved. Major online herbarium databases, including **P** (https://science.mnhn.fr/institution/mnhn/search), **E** (http://www.rbge.org.uk/), **K** (https://www.kew.org/), **A** (https://huh.harvard.edu/), **PE** (https://pe.ibcas.ac.cn/index.html) and Chinese Virtual Herbarium (https://www.cvh.ac.cn/), et al., were checked. The only recorded specimens of *D.subpalmatinervis* stored at **P** were affirmed and checked, utilizing high-resolution digital images of the type specimen. In addition, the authors observed and recorded morphological characters of *Petrocodonlithophilus* in the field and examined its type specimen at **KUN**.

## ﻿Results

### ﻿Comparison and discussion of morphological characteristics of sect. Heteroboea

**Figure 1.**
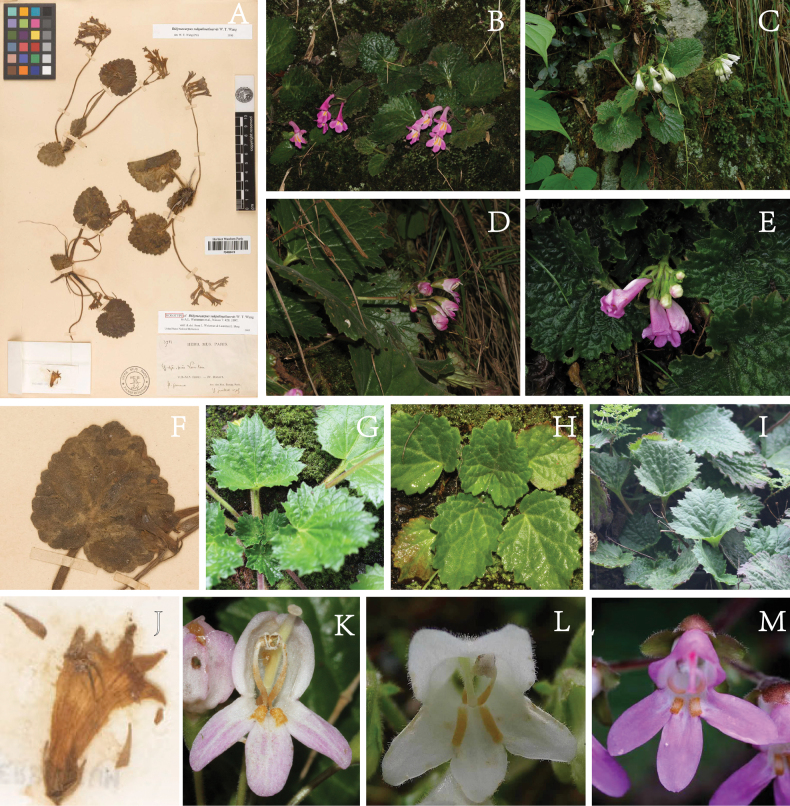
Morphological comparisons of *Didymocarpussubpalmatinervis* and sect. Heteroboea**A** type specimen of *D.subpalmatinervis***B** habit of D.heucherifoliusvar.yinzhengii**C** habit of *D.cortusifolius***D** habit of *D.yuenlingensis***E** habit of D.heucherifoliusvar.gamosepalus**F** leaves of *D.subpalmatinervis***G** leaves of D.heucherifoliusvar.yinzhengii**H** leaves of *D.lobulatus***I** leaves of *D.heucherifolius***J** opened corolla of *D.subpalmatinervis*, showing lobes and stamens **K** frontal view of *D.yuenlingensis*, showing lobes and stamens **L** frontal view of *D.cortusifolius*, showing lobes and stamens **M** frontal view of *D.sinoprimulinus*, showing lobes and stamens.

There are numerous distinct morphological characteristics distinguishing *Didymocarpussubpalmatinervis* from other species within sect. Heteroboea, such as leaves’ margins obtusely or doubly dentate (*vs.* margin irregularly triangular denticulate), triangular lobes (*vs.* rounded or oblong lobes), straight filaments (*vs.* curving), separate anthers (*vs.* confluent anthers). Detailed morphological comparisons with sect. Heteroboea are provided in Fig. 1. Based on the aforementioned, it becomes evident that *D.subpalmatinervis* is not a species within sect. Heteroboea. Additionally, owing to its stemless habit, it does not align with sect. Didymocarpus either. Consequently, the taxonomic classification of this species falls outside the scope of the *Didymocarpus* genus. Given its overall vegetative traits, it should be reclassified under the genus *Petrocodon*.

### ﻿Comparison and discussion of morphological description with *Petrocodonlithophilus*

**Figure 2.**
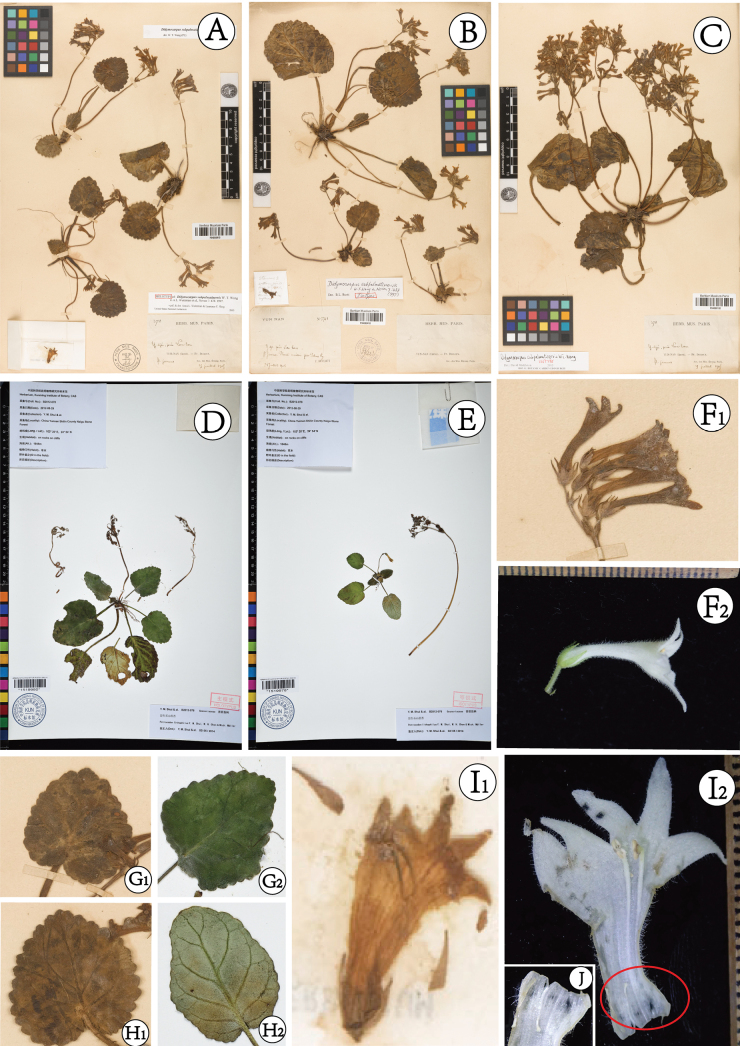
Morphological comparison of *Didymocarpussubpalmatinervis* and *Petrocodonlithophilus***A–C, F_1_–I_1_**: *D.subpalmatinervis***D, E, J, F_2_–I_2_**: *P.lithophilus***A** holotype P04060419 **B** isotype P04060165 **C** isotype P04060418 **D** holotype KUN-1519980 **E** Isotype KUN-1519978 **F** flower **G** abaxial surface of Leaves **H** adaxial surface of Leaves **I** opened corolla **J** staminodes.

Comparing the description of *Didymocarpussubpalmatinervis* and *Petrocodonlithophilus* in their protologues, we found consistency in habit, leaves, calyx, corolla, disc, and ovary (indicated by “●” in Table 1). Because Wen-Tsai Wang described *D.subpalmatinervis* (Weitzman et al. 1997) according to the over 90-year-old type specimen, there may be some distortion compared with wild-living plants in morphology. Moreover, terminology may alter to a certain degree among different taxonomists due to personal preference in the use of words. Taxonomic descriptions have evolved over the years, as have identification tools. For example, Wang selected ‘puberulous’ and Shui picked up ‘pubescent’ to describe the hair of *Didymocarpussubpalmatinervis* and *Petrocodonlithophilus*, respectively. Such equivalents used by them in the description are marked with “★” in Table 1. The only significant difference appeared in the statement of staminode’s number: 2 in *Didymocarpussubpalmatinervis* and 3 in *Petrocodonlithophilus* (indicated by “▲” in Table 1). This feature has already been questioned by Wen et al. (2020). Previous researchers often made mistakes in describing the number of staminodes because 1) they followed generic diagnoses of *Didymocarpus* and paid no attention to the actual stamineal condition; 2) staminodes are inconspicuous in many species unless they are carefully observed, but the correct number of staminodes is 3. Detailed morphological comparisons with *P.lithophilus* are provided in Fig. 2.

**Table 1.** The description and comparison of the *D.subpalmatinervis and P.lithophilus*.

		*Didymocarpussubpalmatinervis* W.T.Wang	*Petrocodonlithophilus* Y.M.Shui, W.H.Chen & Mich.Möller	The similarity in description between the two species
**Habit**		Perennial, stemless herb	Perennial herbs	●
**Leaves**	**shape**	leaf blade ovate to oblate	leaf blade rounded or elliptic	★
	**hair**	adaxially densely appressed puberulous, abaxially appressed puberulous, pilose on veins	pubescent on both surfaces	★
	**base**	cordate	nearly cordate	●
	**margin**	obtusely or doubly dentate	shallowly or deeply crenulate	★
	**apex**	obtuse	rounded	★
**Cymes**	**number**	Cymes ca. 2, ca. 2× branched, 2–10–flowered;	about10 flowers	★
	**peduncle**	puberulous	pubescent	★
	**bracts**	2–3, narrowly lanceolate or linear, puberulous	2, linear or lanceolate, pubescent	★
	**Calyx**	Calyx actinomorphic, 5-sect or nearly so, segments linear-triangular, both surfaces puberulous	Calyx 5-lobed to base, segments linear, pubescent	●
**Corolla**	**color**	Yellow	light greenish-yellow	●
	**length**	2–2.5 cm	2.5–2.8 cm	★
	**tube**	funnelform-cylindric, 1.4–1.7 cm	thin tubular, 1.7–1.9 cm	★
	**lobes**	upper lip 2-lobed, lower lip 3-lobed, all lobes triangular	adaxial lip 2-lobed, lobes triangular, abaxial lip 3-lobed, lobes triangular	●
**Stamens**	**staminodes**	2	3	▲
**Disc**		annular	ring-like	●
**Pistil**	**ovary**	ovary linear	ovary linear inflated	●
	**hairs**	puberulous	pubescent	★
	**stigma**	depressed capitate	stigma 1, disc-like and undivided	★

● Completely consistent characteristics; ★ Overlapping characteristics; ▲ Different characteristics.

The similarity in protologues prompts us to reconsider the circumscription of *Didymocarpussubpalmatinervis* and *Petrocodonlithophilus*.

### ﻿Locality

In 1905, Ducoux collected *Didymocarpussubpalmatinervis* at the position of Y-dje, near Lou-lan. Lou-lan is the French pronunciation of 路南 (Lùnán) in Chinese, and Y-dje corresponds to 维则 (Wéizé). The scope of Lou-lan is not indicated on the type specimens of *D.subpalmatinervis*. China in 1905 was still in the era of the Qing Dynasty. Lunan then refers to Lunanzhou, which is now Shilin County. Naigu Stone Forest is included in Lunan County (now Shilin County). Lunan refers to Lunan Stone Forest. Lunan Stone Forest and Naigu Stone Forest are both in the Stone Forest scenic spot today. Our field survey shows only one species of Gesneriaceae with similar morphological characteristics in these two regions. The distribution of the two species thus overlaps no matter which geographical entity Lunan refers to in the past or present.

In summary, based on literature research, geographical distribution, and morphological analysis, we find no discontinuities and recognize *Didymocarpussubpalmatinervis* and *Petrocodonlithophilus* as conspecific. Therefore, a new combination, *P.subpalmatinervis* (W.T.Wang) F.Wen & Z.L.Li, comb. nov. is proposed here.

### ﻿Taxonomic treatment

#### ﻿ Petrocodon subpalmatinervis

Taxon classificationPlantaeLamialesGesneriaceae

(W.T.Wang) F.Wen & Z.L.Li comb. nov.

E9D30813-E30C-566E-B668-166E99E6D4A3

urn:lsid:ipni.org:names:77326781-1

Didymocarpus subpalmatinervis W.T.Wang, *Novon* 7(4): 428–429. 1997. Type: China. Yunnan: Y-dje, near Lou-lan, July 1905, *Fr. Ducloux 3711* (P: holotype P04060419!; isotypes P04060165!, P04060418!). Basionym.
= Petrocodonlithophilus Y.M.Shui, W.H.Chen & Mich.Möller, *Sys. Bot.* 39(1): 325. 2014. syn. nov. Type: China. Yunnan: Shilin County, Naigu Stone Forest, alt. 1848 m, on rocks on cliffs, 29 August 2012, *Y.M. Shui et al. B2012–078* (**KUN**: holotype KUN-1519980!; isotype KUN-1519978!).

##### Chinese Vernacular name.

掌脉石山苣苔 (Zhǎng Mài Shí Shān Jù Tái).

##### Distribution and habitat.

This species is endemic to Shilin County, Yunnan Province, China, growing in narrow cracks on rocks. Accompanying plants include other shade herbs and trees.

##### Proposed IUCN conservation status.

Naigu Stone Forest is a famous scenic spot for many tourists. The population of *Petrocodonsubpalmatinervis* is easily affected by human activities. For example, tourists probably collect the conspicuous flowers of *P.subpalmatinervis* while they visit the scenic spot and walk along the trails among the Karst peaks and hills of Naigu Shilin. As mature individuals are easily damaged, the population will likely gradually decline year by year. According to the results of our field investigation in the type locality and adjacent regions, the EOO and AOO of *P.subpalmatinervis* are about 800 km^2^ and 30 km^2^. Despite the severe drought in the second half of 2022 seriously influencing the plant population, there is still a high number of individuals (more than 5 000) surviving in the scenic spot. In addition, authors also found several small populations (total>1 000) in the Karst landscape surrounding the type locality, Naigu Shilin. Following the *IUCN Red List Categories and Criteria* (IUCN 2022), this species is evaluated as Endangered [EN B1ab (i, ii, iii, iv)+2ab (i, ii, iii, iv)].

##### Notes.

When Wang published the protologue of *Didymocarpussubpalmatinervis*, the collection date read 1909. But, on the website of **CVH** and the herbarium **P**, the collection date of the information of the specimen we consulted was 1905. Their two different collection years are confusing. We carefully compared the labels of three type specimens of *D.subpalmatinervis* with the collection number ‘*3711*’ and found that the collector’s writing habits might have caused this misunderstanding. Ducloux’s personal collection number increased with time like many collectors. For example, the collection number of *Berberisbodinieri* H.Lév. he collected in 1896 was ‘*0004*’; in 1904, the collection number of *Potamogetondistinctus* A. Benn. was ‘*2571*’; in 1905, he collected *Cornuscapitata* Wall. at the same place (Y-dje near Lou-lan) with the collection number ‘*3715*’; In 1909, the collection number of *Merremiayunnanensis* (Courchet & Gagnep.) R.C.Fang has reached ‘*6398*’. Therefore, it seems likely that the specimens of *D.subpalmatinervis* with the collection number *Fr. Ducloux 3711* were collected in 1905.

For some species published decades or even a hundred years ago, obtaining molecular materials for systematic analysis is challenging because there may be only one type specimen or a few specimens, or they are stored in a foreign herbarium (Wei 2018; Kong et al. 2021). Despite this complicated background, it is still reasonable and accurate to conduct an analysis and verification of the type specimen of the dubious species. When combined with the collecting history of the Ducloux, and the current situation of the existing population (Wei et al. 2022), we are confident about the name and status.

## Supplementary Material

XML Treatment for Petrocodon subpalmatinervis

## ﻿Additional information

### Author ORCIDs

Zheng-Long Li https://orcid.org/0009-0009-8988-5725

Zhang-Jie Huang https://orcid.org/0000-0002-2288-6952

Da-Wei Chen https://orcid.org/0000-0002-8718-8768

Xin Hong https://orcid.org/0009-0003-9477-2414

Fang Wen https://orcid.org/0000-0002-3889-8835

### Data availability

All of the data that support the findings of this study are available in the main text.
